# Experienced Demand Does Not Affect Subsequent Sleep and the Cortisol Awakening Response

**DOI:** 10.2147/NSS.S231484

**Published:** 2020-07-30

**Authors:** Greg J Elder, Mark A Wetherell, Thomas V Pollet, Nicola L Barclay, Jason G Ellis

**Affiliations:** 1 Northumbria Sleep Research Laboratory, Northumbria University, Newcastle Upon Tyne, UK; 2 Department of Psychology, Northumbria University, Newcastle Upon Tyne, UK; 3 Sleep and Circadian Neuroscience Institute, Nuffield Department of Clinical Neurosciences, University of Oxford, Oxford, UK

**Keywords:** stress, cortisol, polysomnography, sleep, anticipation

## Abstract

**Purpose:**

Stress is associated with subjective and objective sleep disturbances; however, it is not known whether stress disrupts sleep and relevant physiological markers of stress immediately after it is experienced. The present study examined whether demand, in the form of cognitive tasks, disrupted sleep and the cortisol awakening response (CAR), depending on whether it was experienced or just anticipated.

**Participants and Methods:**

Subjective and objective sleep was measured in 22 healthy adults on three nights (Nights 0–2) in a sleep laboratory using sleep diaries and polysomnography. Saliva samples were obtained at awakening, +15, +30, +45 and +60 minutes on each subsequent day (Day 1–3) and CAR measurement indices were derived: awakening cortisol levels, the mean increase in cortisol levels (MnInc) and total cortisol secretion (AUC_G_). On Night 1, participants were informed that they were required to complete a series of demanding cognitive tasks within the sleep laboratory during the following day. Participants completed the tasks as expected or unexpectedly performed sedentary activities.

**Results:**

Compared to the no-demand group, the demand group displayed significantly higher levels of state anxiety immediately completing the first task. There were no subsequent differences between the demand and no-demand groups in Night 2 subjective sleep continuity, objective sleep continuity or architecture, or on any Day 3 CAR measure.

**Conclusion:**

These results indicate that sleep and the CAR are not differentially affected depending on whether or not an anticipated stressor is then experienced. This provides further evidence to indicate that the CAR is a marker of anticipation and not recovery. In order to disrupt sleep, a stressor may need to be personally relevant or of a prolonged duration or intensity.

## Introduction

Stress has long been associated with disturbances to both subjective and objective sleep,^1^,^2^ and naturalistic studies have indicated that the anticipation of upcoming stress can disrupt subjective and objective sleep.^3^,^4^ A recent laboratory study demonstrated that anticipated stress, in the form of next-day demand, did not affect subjective or objective sleep.^5^ However, it is not known if stress can cause a “rebound” effect, whereby sleep is disrupted immediately (i.e. during the subsequent night) after the anticipated stressful event is then experienced.

Cortisol, which is the end product of the hypothalamic-pituitary-adrenal (HPA) axis, is responsive to psychological demand in a dose-response manner^6^ and is a suitable physiological marker of stress. The cortisol awakening response (CAR), which refers to the sharp increase in cortisol levels (of between approximately 38–75%) which are observed in response to awakening, has been shown to be sensitive to anticipated demand.^5^,^7^ However, the CAR may also function as a marker of recovery from previous demand^8^ and is therefore an suitable physiological marker of assessing whether experiencing an anticipated stressor can disrupt the HPA axis.

The aim of the present study was to investigate whether an anticipated stressor subsequently disrupted subjective and objective sleep, and the CAR. Following the anticipation of a demanding day, it was expected that those who experienced demand would demonstrate poorer subjective and objective sleep, and an altered CAR profile, compared to those who did not.

## Participants and Methods

### Participants

Twenty-two healthy participants (M_age_ = 23.42 years; SD_age_ = 3.62 years, 50% male, 50% female) were recruited from the staff and student population of Northumbria University. Participants provided written informed consent and were paid £150 upon completion of the study.

Full screening procedures and detailed demographic information are reported in detail elsewhere.^5^ Briefly, participants were screened for current or previous sleep problems, physical or psychiatric illnesses, shift work or trans-meridian travel in the three months prior to study enrolment, using a structured clinical interview with a member of the sleep laboratory staff. Participants were not permitted to take part if there was any evidence of sleep difficulties or of current or previous physical/psychiatric illness. In addition, participants completed self-reported sleep diaries and two weeks of actigraphy prior to attending the sleep laboratory for the overnight part of the study. These were visually inspected by sleep laboratory staff in order to verify that sleep/wake schedules were stable prior to participation.

After consenting, participants were allocated to a demand (n = 11) or no-demand (n = 11) group. The demand group was intentionally recruited and completed the study before the no-demand group. This was a deliberate decision in order to ensure that the demand group did not reveal the true purpose of the study to the no-demand group in advance, due to the study population.

### Procedure

**Figure 1:**
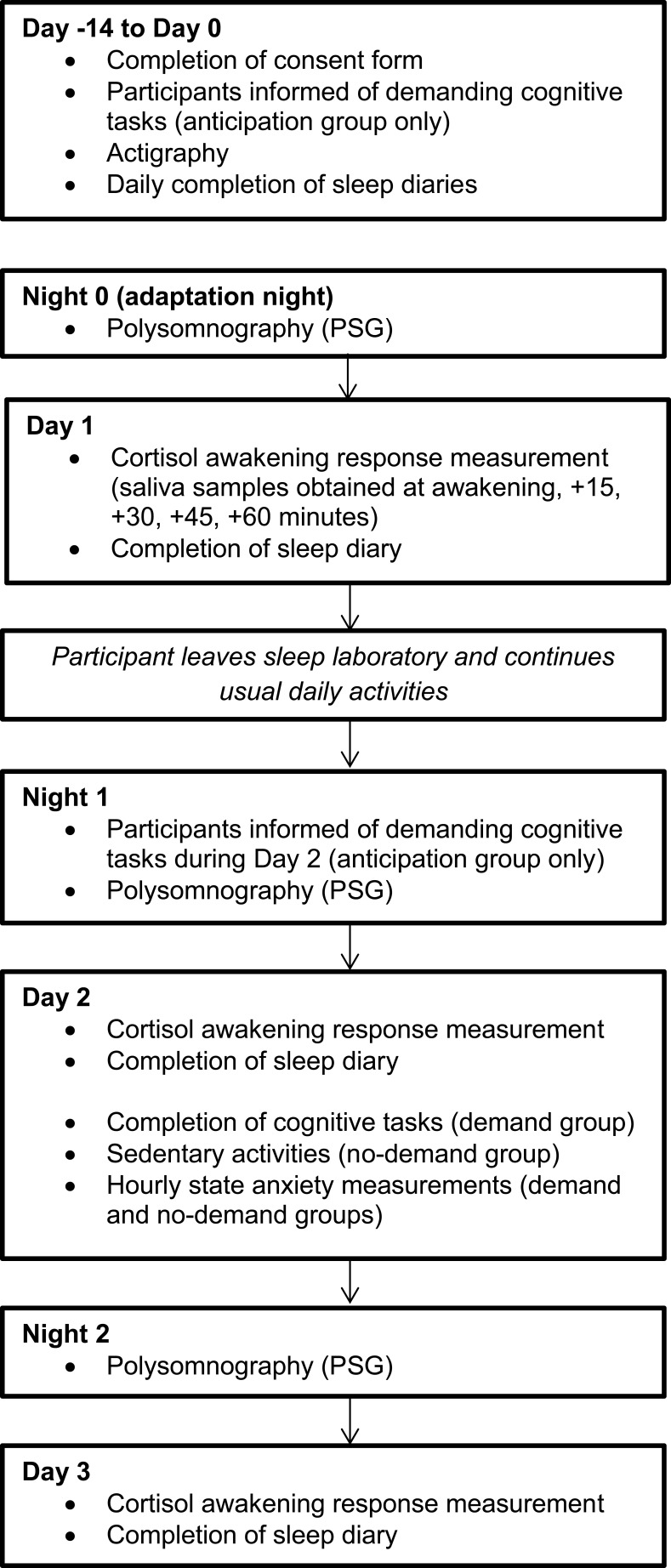
Study schematic.

The procedure has been previously described in detail elsewhere^5^,^9^ and is summarised in Figure 1. Participants provided informed consent and were confirmed as being a healthy good sleeper by assessing their sleep, psychiatric and physical illness history as described above. Participants also completed the Pittsburgh Sleep Quality Index (PSQI^10^) and Hospital Anxiety and Depression Scale (HADS^11^) as a measure of sleep quality, and of subjective anxiety and depression.

Participants completed a baseline period where sleep was monitored using sleep diaries and actigraphy (Days −14 to 0) before sleeping for three consecutive weekday nights in a sleep laboratory (Nights 0–2). Lights out and wake-up times were scheduled in accordance with habitual (baseline sleep diary) times. Participants left the laboratory on Day 1, returned on Night 1, and remained under observation in the sleep laboratory until Day 3.

Sleep diaries^12^ were used to measure subjective sleep continuity (total sleep time (TST), time in bed (TIB), sleep efficiency (SE%: (TST/TIB × 100)), sleep-onset latency (SOL), number of awakenings (NWAK) and wake after sleep onset (WASO)) and polysomnography (PSG) was used to measure objective sleep. Mastoid and ground-linked EEG electrodes were placed at FP_1_, FP_2_, F_3_, F_4_, C_3_, C_4_, P_3_, P_4_, O_1_, O_2_ and C_z_ and recordings were externally blind-scored in accordance with standard guidelines.^13^,^14^ For measurement of the CAR, saliva samples were collected at awakening, +15, +30, +45 and +60 minutes on three consecutive mornings (Day 1 – Day 3) using Salivettes (Sardstedt, Leicester, UK).

On Night 1, all participants were informed that they would remain in the sleep laboratory during Day 2 in order to complete a range of demanding cognitive tasks, where the best performance on a randomly-chosen task would be rewarded with a prize in order to elicit competition and arousal. On Day 2 from wake +3hrs to +13hrs, the participants in the demand condition (n = 11) completed hourly computerised tasks of 10–15 minutes in duration (Emotional Stroop task,^15^ Multi-Tasking Framework^16^ and Iowa Gambling Task^17^). As a measure of state anxiety, all participants responded to statements from the short-form state anxiety scale,^18^ using 100mm visual analogue scales, where 0mm indicated “not at all” and 100mm indicated “very much”. State anxiety was measured at wake +60 minutes, and hourly thereafter, except at meal breaks (provided at wake +2hrs, +6hrs and +10hrs).

On Day 2 the no-demand group (n = 11) was informed that they were not required to complete any tasks, and instead remained in the sleep laboratory performing sedentary activities including reading and watching television.

### Data Analysis

Night 2 subjective and objective sleep, and Day 3 CAR data are reported. Measures of subjective sleep continuity (TIB, TST, SE%, SOL, NWAK and WASO), objective sleep continuity (TST, SE%, SOL, NWAK and WASO) and objective sleep architecture (percentages of sleep spent in REM, N1, N2 and N3) were compared between groups using *t*-tests adjusted for multiple comparisons (adjusted *p*-values = 0.008, 0.013 and 0.006).

CAR data from five participants (demand n = 2; no demand n = 3) were excluded due to saliva samples containing an insufficient volume of saliva for analysis. The CAR was examined by comparing cortisol levels (nanomoles per litre; nmol/l) between groups using a 2 × 5 mixed analysis of variance (ANOVA). Additional CAR indices were compared between groups using *t*-tests: awakening cortisol levels, the mean increase in cortisol levels during the measurement period (MnInc^19^) and total cortisol secretion, expressed as the area under the curve with respect to ground (AUC_G_), adjusted for multiple comparisons (adjusted *p*-value = 0.017). Effect sizes are reported using Cohen’s *d*. State anxiety was compared between groups using a 2 × 10 mixed ANOVA, with follow-ups adjusted for multiple comparisons (adjusted *p*-value = 0.005).

## Results

Participant sleep quality was within the normal range (PSQI *M* = 3.36, *SD* = 1.89), as was subjective anxiety (HADS Anxiety *M* = 5.41, *SD* = 3.17) and subjective depression (HADS Depression sub-scale *M* = 2.36, *SD* = 1.97).

**Figure 2:**
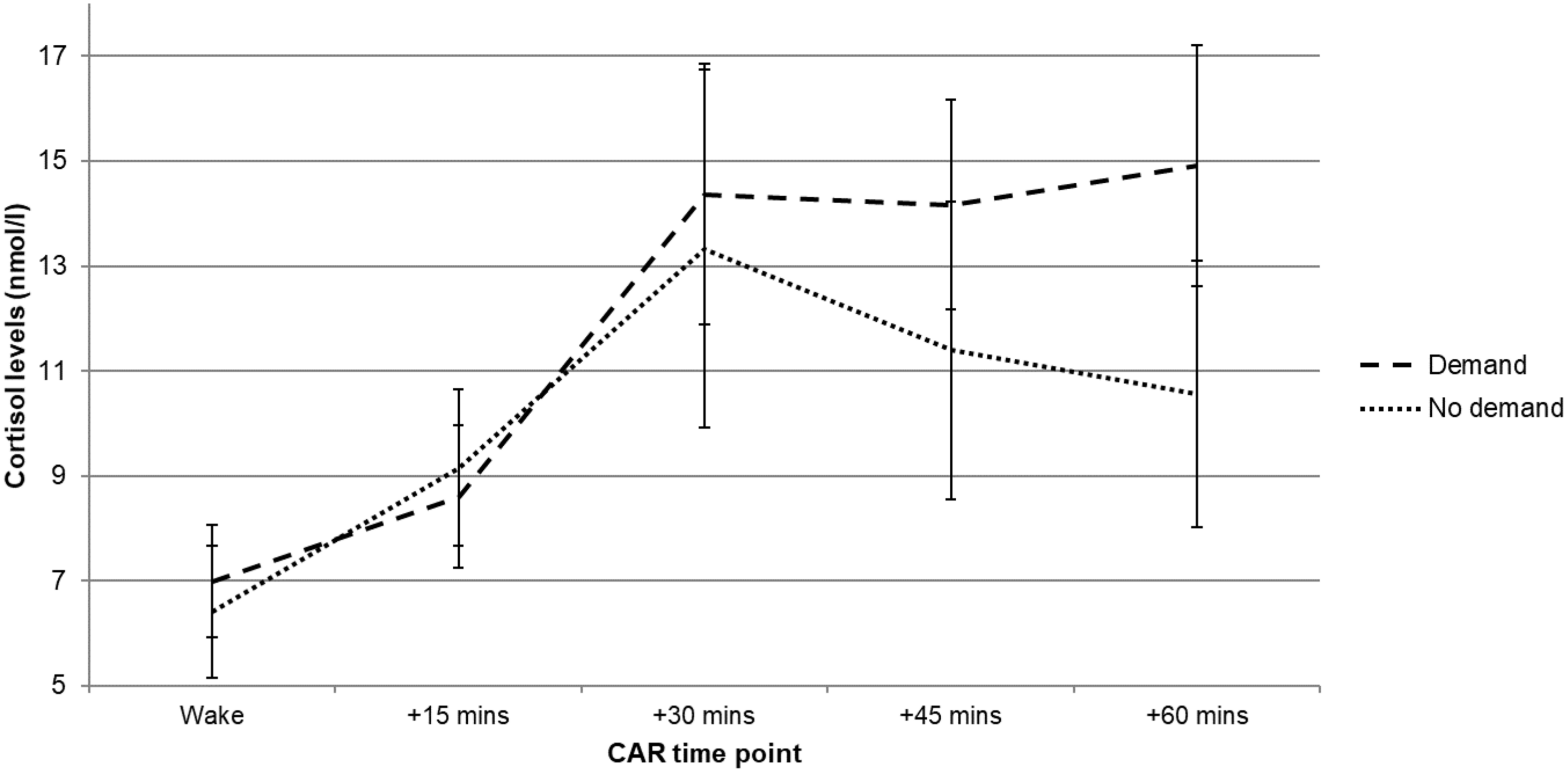
Day 3 mean (±SEM) CAR profile comparisons between demand and no-demand groups. There were no significant differences (*p*-values > 0.05).

There were no between-group differences in any subjective or objective measure of sleep continuity or architecture (*p*-values > 0.05; Tables 1 and 2). Cortisol levels showed a main effect of time point (*F*(2.52, 37.74) = 0.94, *p <*0.001, η2_p_ = 0.06; Figure 2), representing a typical increase in cortisol levels during the CAR measurement period. The time point × group interaction, and main effect of group, was not significant (*p*-values > 0.05). There were no significant between-group differences in awakening cortisol levels, MnInc, or total cortisol secretion (*p*-values > 0.05; Table 3).

**Table 1:** Night 2 Subjective Sleep Continuity Comparisons

	Demand (n = 11)		No Demand (n = 11)		*p*-value	Effect Size (*d*)
	Mean	SD	Mean	SD		
TIB (mins)	532.27	42.80	539.55	44.52	0.700	0.17
TST (mins)	455.82	60.45	470.27	40.04	0.516	0.30
SOL (mins)	21.36	16.45	10.68	5.25	0.063	0.92
NWAK	1.00	0.89	1.18	1.15	0.683	0.18
WASO (mins)	5.82	6.66	3.36	5.84	0.369	0.41
SE (%)	85.44	7.70	87.20	3.27	0.495	0.31

**Abbreviations:** TIB, time in bed; TST, total sleep time; SOL, sleep onset latency; NWAK, number of awakenings; WASO, wake after sleep onset; SE, sleep efficiency.

**Table 2:** Night 2 Demand and No-Demand Group Objective Sleep Comparisons

	Demand (n = 11)		No Demand (n = 11)		*p*-value	Effect Size (*d*)
	Mean	*SD*	Mean	*SD*		
TST (mins)	443.00	39.48	456.86	40.48	0.426	0.36
SOL (mins)	13.23	10.25	8.27	5.40	0.176	0.64
NWAK	12.73	4.34	10.91	4.44	0.343	0.43
WASO (mins)	12.45	10.22	9.55	9.17	0.490	0.31
SE (%)	94.55	2.74	96.21	2.13	0.128	0.71
Time in REM (%)	23.86	6.32	24.08	7.33	0.941	0.03
Time in N1 (%)	3.74	1.76	2.62	1.46	0.121	0.73
Time in N2 (%)	51.48	9.13	51.19	7.03	0.934	0.04
Time in N3 (%)	20.90	6.66	22.10	5.67	0.654	0.20
Latency to REM (mins)	102.27	57.93	95.00	46.38	0.749	0.15
Latency to N1 (mins)	13.23	10.25	8.27	5.40	0.176	0.64
Latency to N2 (mins)	21.14	9.88	13.68	7.24	0.057	0.90
Latency to N3 (mins)	33.95	14.00	26.23	9.96	0.151	0.67

**Abbreviations:** TST, total sleep time; SOL, sleep onset latency; NWAK, number of awakenings; WASO, wake after sleep onset; SE, sleep efficiency; REM, rapid eye movement sleep; N1, stage 1 sleep; N2, stage 2 sleep; N3, stage 3 sleep.

**Table 3:** Day 3 Additional Cortisol Awakening Response Measurement Indices by Group

	Demand (n = 9)		No Demand (n = 8)		*p*-value	Effect Size (*d*)
	Mean	*SD*	Mean	*SD*		
Awakening levels (nmol/l)	7.00	3.79	6.41	3.04	0.731	0.18
AUC_G_ (nmol/l)	721.30	396.51	635.44	258.62	0.610	0.27
MnInc (nmol/l) (arbitrary units)	6.02	5.58	4.70	3.43	0.574	0.30

**Abbreviations:** AUC_G_, area under the curve with respect to ground; MnInc, mean increase.

**Figure 3:**
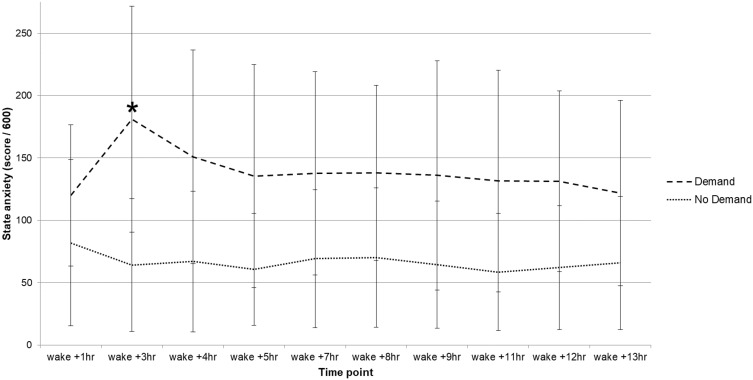
Day 3 state anxiety levels between demand and no-demand groups (*p < 0.005).

For state anxiety, the main effect of time point was not significant (*p* > 0.05). There was a significant main effect of condition (*F*(1, 19) = 6.68, *p* = 0.018, η2_p_ = 0.26, and a significant time point × group interaction (*F*(4.06, 77.17) = 2.51, *p* = 0.048, η2_p_ = 0.12. Follow-up comparisons indicated that the demand group showed significantly higher levels of state anxiety at wake +3hrs (*p* = 0.003), occurring immediately after the demand group completed the first task (Figure 3).

## Discussion

This study investigated whether an anticipated stressor subsequently disrupted subjective sleep, objective sleep, and the CAR. There were no subsequent differences in subjective or objective sleep between participants who experienced demand and participants who did not experience demand. This indicates that an anticipated stressor does not cause a disruptive “rebound” effect upon sleep following the stressor. There were no statistically significant between-group differences in the CAR of the following day. There was, however, a trend towards increased cortisol levels from wake to +30 minutes in the demand group and this is likely to indicate that there are high levels of individual differences in the CAR following an anticipated stressor. In order to confirm whether or not this is the case with the CAR, these findings should be replicated and investigated further in a larger sample.

The demand placed upon participants occurred repeatedly at multiple time points throughout the day, was of a greater duration than the anticipated demand in previous stress-induction studies^20^ and demonstrably increased levels of state anxiety in comparison to participants who did not experience the demand. However, it is still possible that a stressor may need to be of a greater intensity, severity or duration, or that a stressor may need to be personally-relevant, in order to disrupt subjective or objective sleep. Alternatively, the advance warning of the demand may have allowed participants to prepare and therefore mitigate the impact upon sleep, as is suggested by the CAR results in the present study.

A particular strength of the study is in the highly-controlled sleep laboratory environment, which ensured complete control over relevant environmental factors including light levels, participant food intake and exercise, as well as ensuring accurate saliva sample collection.^21^ This is important since short delays to sample collection can lead to an inaccurate CAR.^22^ A limitation is in the relatively small sample size, and although the high level of control offers advantages over ambulatory studies, these findings should still be considered to be preliminary and should be replicated with larger samples.

## Conclusions

Overall, an anticipated stressor does not disrupt objective sleep in the subsequent night, or the CAR during the subsequent day; this indicates that the CAR is not a marker of recovery^8^ and is instead a marker of anticipation.^5^,^7^ In order to disrupt sleep, the stressor may need to be personally relevant, or of a longer duration or intensity.

## Abbreviations

ANOVA, analysis of variance; AUC_G_, area under the curve with respect to ground; CAR, cortisol awakening response; EEG, electroencephalography; EMG, electromyography; MnInc, mean increase; N1, non-rapid eye movement stage 1; N2, non-rapid eye movement stage 2; N3, non- rapid eye movement stage 3; NWAK, number of awakenings; PSG, polysomnography; REM, rapid eye movement; SE, sleep efficiency; SOL, sleep onset latency; TIB, time in bed; TST, total sleep time; WASO, wake after sleep onset.
